# Role of intrarenal complement production in kidney transplantation

**DOI:** 10.1093/ckj/sfaf135

**Published:** 2025-05-01

**Authors:** Paolo Molinari, Shivani Wadnerkar, Katja L Ferrari, Giuseppe Castellano, Nicholas Chun, Paolo Cravedi

**Affiliations:** Translational Transplant Research Center and Department of Medicine, Icahn School of Medicine at Mount Sinai, New York, NY, USA; Unit of Nephrology, Dialysis and Kidney Transplantation, Fondazione IRCCS Ca' Granda Ospedale Maggiore Policlinico di Milano, Milan, Italy; Translational Transplant Research Center and Department of Medicine, Icahn School of Medicine at Mount Sinai, New York, NY, USA; Department of Pediatrics, Icahn School of Medicine at Mount Sinai, New York, NY, USA; Translational Transplant Research Center and Department of Medicine, Icahn School of Medicine at Mount Sinai, New York, NY, USA; Unit of Nephrology, Dialysis and Kidney Transplantation, Fondazione IRCCS Ca' Granda Ospedale Maggiore Policlinico di Milano, Milan, Italy; Translational Transplant Research Center and Department of Medicine, Icahn School of Medicine at Mount Sinai, New York, NY, USA; Translational Transplant Research Center and Department of Medicine, Icahn School of Medicine at Mount Sinai, New York, NY, USA

**Keywords:** acute rejection, chronic rejection, glomerular disease, organ transplant, tubular disease

## Abstract

Systemic complement is a major contributor to the onset and progression of kidney graft injury. However, the kidney itself is an important site of complement production. Renal-derived complement plays a key role in graft dysfunction, unlike in some other solid organ transplants. Complement factors are generated by multiple renal cell types under both physiological and pathological conditions. Renal complement production mediates ischemia/reperfusion injury and acute cellular and humoral rejection but protective effects of the complement cascade have been reported as well. More recently, intracellular complement production and activation (complosome) has also been shown to be an important regulator of key metabolic and cellular functions in renal cells and in immune kidney infiltrates, adding complexity to the field.

Herein, we review current knowledge on the role of renal-derived complement in the pathophysiology of kidney graft damage and the current landscape of complement targeted therapeutics in kidney transplantation.

## INTRODUCTION

Kidney transplantation is the ideal treatment for patients with end-stage kidney disease, as it confers significant survival benefits compared to long-term dialysis [1]. Despite promising advances in early graft survival, long-term outcomes are still disappointing [2, 3]. Significant evidence implicates the complement system in the pathogenesis of renal graft failure. Upregulation and activation of both systemic and locally produced complement promotes ischemia-reperfusion injury (IRI), cellular rejection, and antibody-mediated rejection [4]. While systemic complement has traditionally been considered the pathogenic mediator, newer evidence has highlighted the critical role of local kidney-derived complement.

Here, we review the current understanding of the role of intrarenal complement production in the establishment and progression of kidney graft damage.

### Complement pathway: overview of function, activation, and regulation

The complement system is a complex network composed of >30 soluble and membrane-bound proteins that interact to protect the body against infection, facilitate debris removal, and bolster adaptive immunity [5]. Systemically circulating complement proteins are mostly produced by the liver, but local complement production occurs in several other organs [6]. Importantly, the kidney is a significant site of extrahepatic complement production in the setting of transplantation [7, 8].

**Figure 1:**
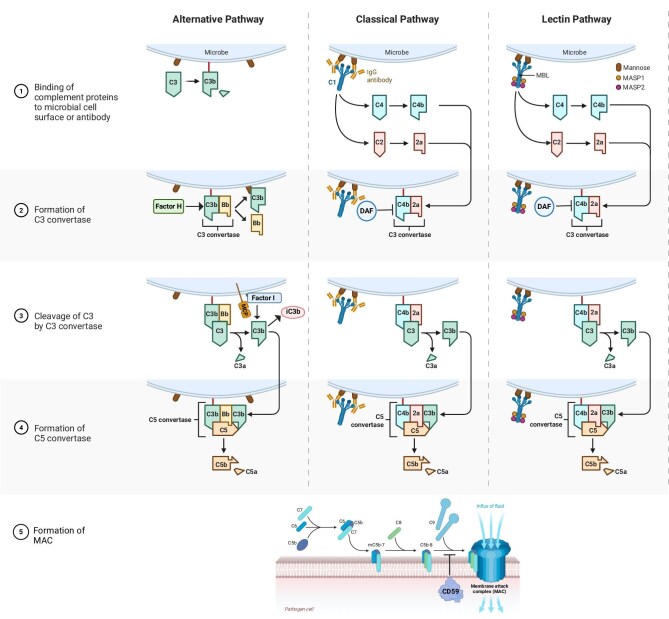
Overview of complement activation pathways, effector functions, and inhibitors. C1q, r, s cross-linking of antibodies activates the CP. MASPs bind to mannose motifs expressed on bacteria to activate complement via the lectin pathway, while the AP kicks off with its “tick-over” process, i.e. spontaneous hydrolysis of C3. The three pathways form C3 convertases that cleave C3 into C3a and C3b, amplifying complement activation. The addition of a second C3b molecule to either C3 convertase forms the C5 convertase, which cleaves C5 into C5a and C5b. C5b, in conjunction with C6 to C9, comprises the MAC. C3b functions as an opsonin, whereas C3a and C5a have inflammatory and chemotactic properties. DAF (CD55) and MCP (CD46) are cell surface-expressed complement regulators that accelerate the decay of all surface-assembled C3 convertases, thereby limiting the amplification of the downstream cascade. MCP and Factor H also have cofactor activity: in conjunction with soluble Factor I, they irreversibly cleave C3b to iC3b, thereby preventing re-formation of the C3 convertase. CD59 inhibits formation of the MAC.

The complement cascade can be activated through three pathways: the classical pathway (CP), the mannose-binding lectin (MBL) pathway (LP), and the alternative pathway (AP) (Fig. 1). The CP is initiated when C1q protein recognizes antigen-bound IgG or IgM (immunoglobins) antibodies, inducing a conformational shift and formation of a C1qrs complex that then cleaves C4 and C2 to generate a C4bC2b C3-convertase. The LP similarly generates C4bC2b convertases but is initiated by MBL, which recognizes pathogen-specific carbohydrate motifs (e.g. mannose) as well as autologous proteins expressed by apoptotic and necrotic cells [9, 10]. Surface-bound MBL recruits mannose-associated serine proteases (MASPs) to cleave C4 and C2 as above. In contrast to the CP and LP, which require recognition of an antigenic target, the AP spontaneously activates at low levels. This “tick-over” mechanism non-specifically surveils the microenvironment, with healthy host cells expressing regulatory proteins that impede pathogenic complement deposition and activation.

All three pathways converge to form C3 convertases, multimeric enzymatic complexes that cleave C3 into C3a and C3b. These enzymes form the backbone of the central feed-forward amplification loop. Cell surface-bound C3b can further form C5 convertases that split C5 into C5a and C5b. C5b binds complement components C6–9 to form the membrane attack complex (MAC) which inserts itself in target cell membranes to form a lytic pore. The soluble split products, C3a and C5a, amplify inflammation by directly activating immune cells and serving as potent chemoattractants, while surface-bound opsonins, including C3b, iC3b, and C3dg, promote phagocytosis by innate immune cells such as macrophages and neutrophils [4, 5, 11].

Complement activation is tightly regulated by membrane-bound and soluble proteins to prevent damage to bystander self-cells [12]. Decay-accelerating factor (DAF or CD55) is a glycophosphatidylinositol-anchored complement regulator that accelerates the decay of cell surface-bound C3 convertases. Thus, DAF limits downstream complement activation and restricts the production of the complement cleavage products [13]. DAF limits complement activation on the cell surfaces where it is expressed, and not on proximal cells (e.g. pathogens, dead cells) that lack DAF expression. In murine models, genetic deletion or reduced expression of DAF resulted in increased complement activation and heightened disease severity in conditions such as membranous nephropathy and autosomal dominant polycystic kidney disease [14, 15]. On the other hand, DAF overexpression in murine models prevented induction of focal segmental glomerulosclerosis and diabetic glomerulosclerosis [16, 17].

Human CD46 (murine homolog CRRY, complement receptor 1-related protein Y), also known as membrane cofactor protein (MCP), has a similar decay-accelerating function, but also exhibits cofactor activity. In conjunction with soluble Factor I, this membrane-bound regulator permanently inactivates C3b to iC3b, thereby preventing the further generation of C3 convertase [18]. Other examples of complement regulators include: CD59 (protectin), a cell surface-expressed regulator that inhibits MAC by preventing polymerization of C9 [19], complement Factor H (CFH), a soluble complement regulatory protein that exhibits both decay-accelerating and cofactor activity [20], and complement receptor 1 (CR1) that limits amplification of the complement cascade and MAC formation by inhibiting C3 convertases [4].

### Intrarenal complement production

The earliest evidence for intrarenal complement production dates back >50 years, when researchers hypothesized that local complement activation could be central to kidney physiology and described complement Factor 4 expression in healthy human kidneys. [21, 22]. Others subsequently demonstrated increased intrarenal expression of genes encoding C2, C3, C4, and Factor B in a murine model of lupus nephritis, suggesting that local renal production of complement components may modulate autoimmune kidney disease [23].

**Figure 2:**
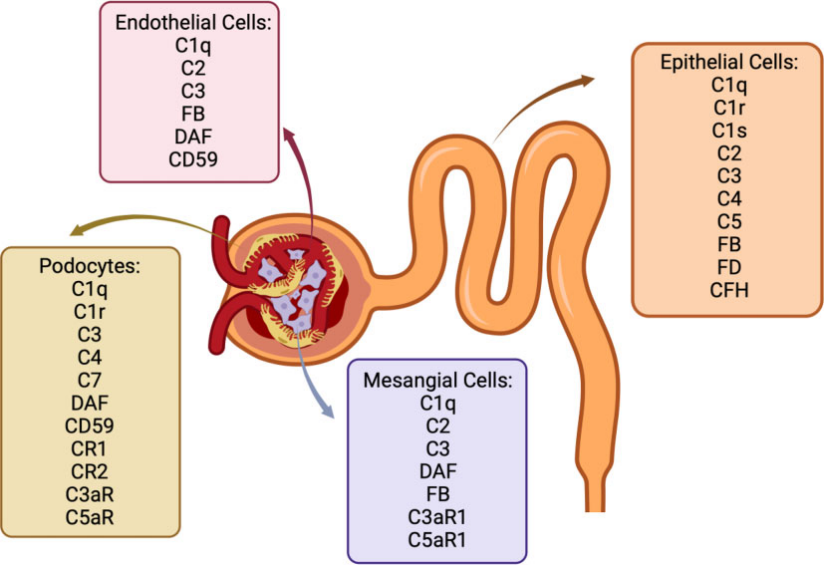
Main complement production sites and expression of complement receptors and regulators within the nephron. Complement components are produced by many cell types in the nephron: endothelial cells, podocytes, mesangial cells, and epithelial cells. All these cells produce C1q and C3. Epithelial cells are the only ones that produce C1r and C1s (and C1q, C2, C3, C4, C5), while mesangial and endothelial cells do not create C4 and C5, and podocytes do not create C2 and C5. All the sections produce two or three complement regulators among the following: FB, FD, DAF, CFH, and CD59. Complement receptors 1 and 2 are located in the podocytes only, but complement 5a and 3a receptors can be found both in the podocytes and mesangial cells. This demonstrates an abundant production of complement factors in kidney cells.

Since these original findings, several studies confirmed that different renal cells can produce complement components (Fig. 2). Renal tubular epithelial cells (TECs) are the predominant source of intrarenal complement production and can produce almost all complement proteins under both physiologic and pathophysiologic conditions [12, 24, 25]. Proximal TECs produce C3 which is increased by the pro-inflammatory cytokines IL-2, IL-1, IFNγ, and IL-17 [26–29]. Generation of complement Factor B, C2, C4, and CFH can also be induced by stimulation with IFN-γ and IL-1 [30–32]. In contrast, anti-inflammatory mediators such as TGF-β have an inhibitory effect on complement production in proximal TECs [33].

Cultured human glomerular mesangial and epithelial cells are also capable of secreting a variety of CP, AP components, and regulatory proteins, namely C3, C4, MCP, DAF, CR1, and CD59 [31, 34–38]. Podocytes also secrete and/or express several complement proteins and regulators. Gene expression of C1q, C1r, C2, C3, C3a receptor (C3aR), C5a receptor (C5aR), C7, CR1, and CR2, have all been demonstrated in cultured podocytes, and increase following podocyte injury [39, 40]. Faseeb *et al.* found that local synthesis of complement protein C3 in the kidney significantly contributes to tissue injury in proteinuria-associated renal disease. Mice lacking local C3 synthesis were protected from tubular damage and renal failure despite normal circulating C3 levels. Histological analysis showed reduced complement activation, inflammation, and fibrosis in C3-deficient kidneys [41, 42]. Still, a recent paper evaluating the role of C5a receptors in tubular injury revealed an unexpected outcome. In this case, C5aR1-deficient mice had exacerbated toxin-induced acute kidney injury compared to wild-type (WT) controls [43], suggesting that injury-induced complement activation in the kidney is may not be universally pathogenic.

### Impact of graft-produced complement on kidney transplantation

#### Complement production and activation in donor organs

Upregulation of renal C3 expression may be a direct consequence of brain death [44, 45], and intrarenal complement expression negatively affects both early and late graft function in kidneys derived from donations after brain death [46, 47]. This holds clinical significance, as an elevation in local C3 expression is linked to inferior graft function following transplantation [48, 49]. Indeed, renal epithelial cells from donation-after-brain-death organs show increased expression of complement receptors such as C5aR1, suggesting a deleterious axis of local complement production and direct signaling on the allograft [50]. This finding is consistent with pre-clinical murine transplant work showing that local complement C3 production modulates renal allograft injury during acute cellular rejection (ACR) and isograft transplant experiments showing that complement C3-deficient kidneys were protected from adriamycin-induced proteinuric kidney damage even if transplanted into C3-sufficient hosts. Pratt *et al.* demonstrated that mice receiving C3-deficient (C3^–/–^) kidney grafts exhibited significantly prolonged graft survival and reduced anti-donor T-cell responses compared to those receiving WT kidneys. Histological analysis confirmed reduced leukocyte infiltration and inflammation in C3^–/–^ grafts. Additionally, the recipients of C3^–/–^ grafts showed impaired T-cell priming, which is consistent with later work that demonstrated that leukocyte infiltration mediated by intrarenal C5a–C5aR interactions increased donor antigen exposure to the recipient's immune system [51].

#### Ischemia-reperfusion-associated kidney complement production

During IRI, complement production by renal endothelial cells, renal tubular cells, and infiltrating immune cells is dramatically upregulated. Kidney transplantation studies that use syngeneic rats (i.e. in the absence of an alloimmune response) demonstrate a burst of C3 mRNA expression within the glomeruli due to ischemic injury [52, 53]. Local activation through the AP results in formation of C3a/C5a, which bind their respective receptors expressed on kidney cells and amplify inflammation [54–58]. Allogeneic mouse transplant studies confirmed that transplant-associated IRI [4] impairs graft function through local complement production by donor TECs, rather than circulating recipient-derived complement components [57].

**Figure 3:**
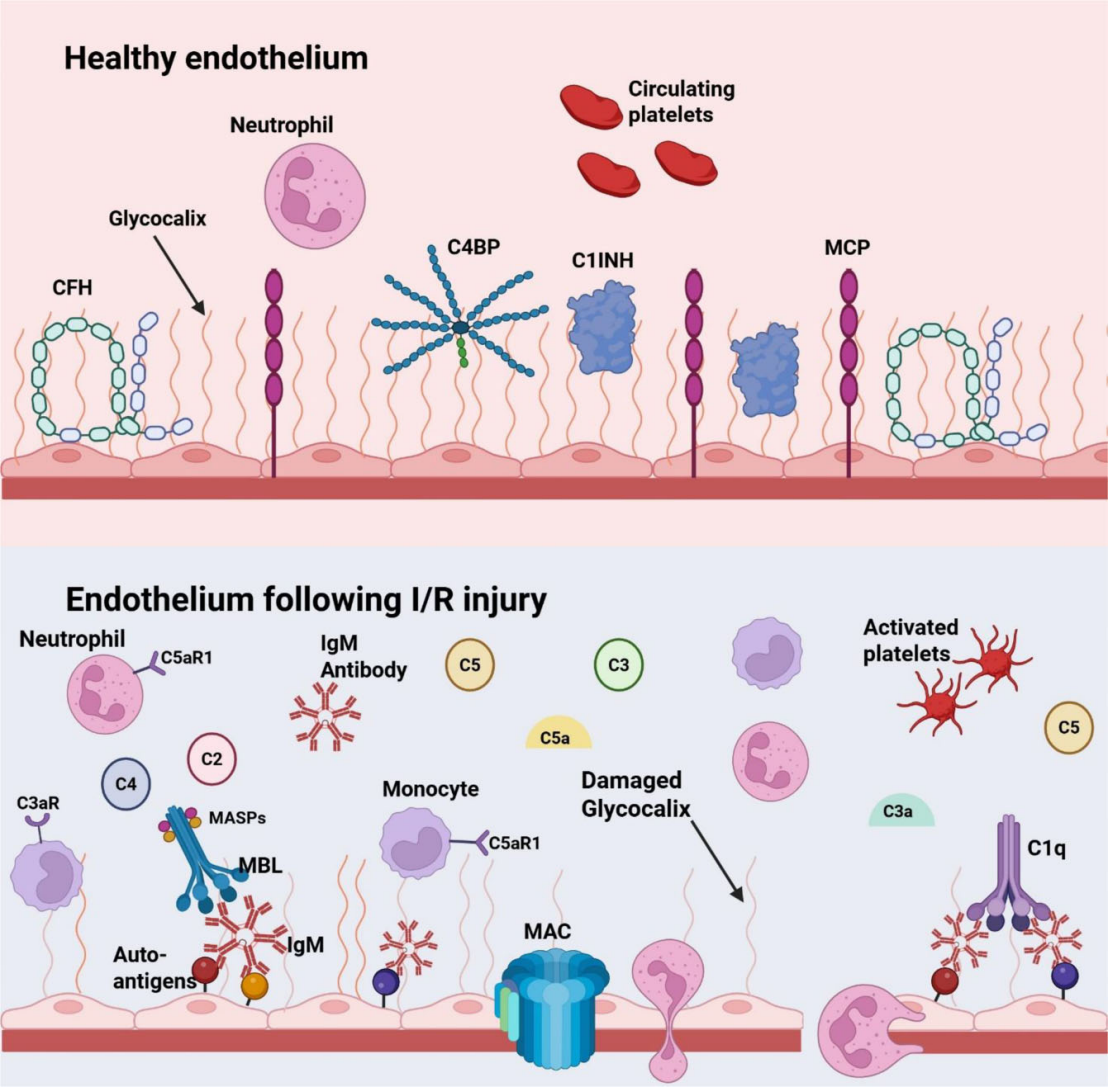
Mechanism of complement-mediated endothelial injury during IRI. The healthy endothelium is protected by the glycocalyx, which binds several regulators of the complement system such as C1INH (C1 inhibitor), CFH, and C4BP (C4-binding protein), together with MCP that inhibits C3 and C5 convertases. On IRI, complement activates though the lectin and classical pathways (natural antibodies bind to newly exposed autoantigens), leading to the formation of MAC, which, together with inflammatory cytokines damage the glycocalyx. C3a and C5a can bind to C3aR (complement 3a receptor), C5aR1 (complement 5a receptor 1), and C5aR2 (complement 5a receptor 2) to stimulate chemotaxis of neutrophils and monocytes to the ischemic region. These receptors are expressed in the kidney, primarily in proximal tubules and podocytes. Furthermore, metalloproteinases and heparanases are released from the endothelial cells and break down the glycocalyx freeing complement regulators and enhancing complement activation and injury. Therefore, in IRI, the complement system activates through the formation of C3 and C5 convertases and is further facilitated by the loss of regulatory proteins in the glycocalyx. Additionally, activated platelets and neutrophils form complexes and complement-induced neutrophil activation favors their migration through the activated endothelium, further aggravating the pro-inflammatory and pro-thrombotic response [110].

In a seminal study, Pratt *et al.* used a rat model of IRI, exposing kidneys to 24 hours of cold ischemia followed by 2 hours of reperfusion, and detected aberrant methylation in the C3 gene promoter region that was associated with increased transcription. Given the potential for epigenetic modifications to stabilize and pass down to progeny cells, they posited that these alterations might lead to excess complement production even beyond the immediate IRI period [59]. Subsequent human studies supported these findings, showing complement gene expression after reperfusion is higher in kidneys from deceased (enhanced IRI) versus living (minimal IRI) donors [47, 60] (Fig. 3).

Altered expression of complement inhibitors within the tubular epithelium also emerges as a critical factor in IRI. *In vitro* inhibition of CRRY expressed by proximal TECs results in AP-mediated injury. Increased C3 mRNA and decreased CFH mRNA were detected in the outer medulla after IRI [61]. The protective role of tubular complement inhibitors was confirmed by experiments treating allo-kidneys with an analog of the human complement regulatory protein CD35/CR1 that blocks C3 convertase. When exposed to prolonged cold ischemia before transplantation, kidneys pretreated with this reagent showed superior graft function, less tubular damage, and less complement activation and deposition compared to controls [62].

**Figure 4:**
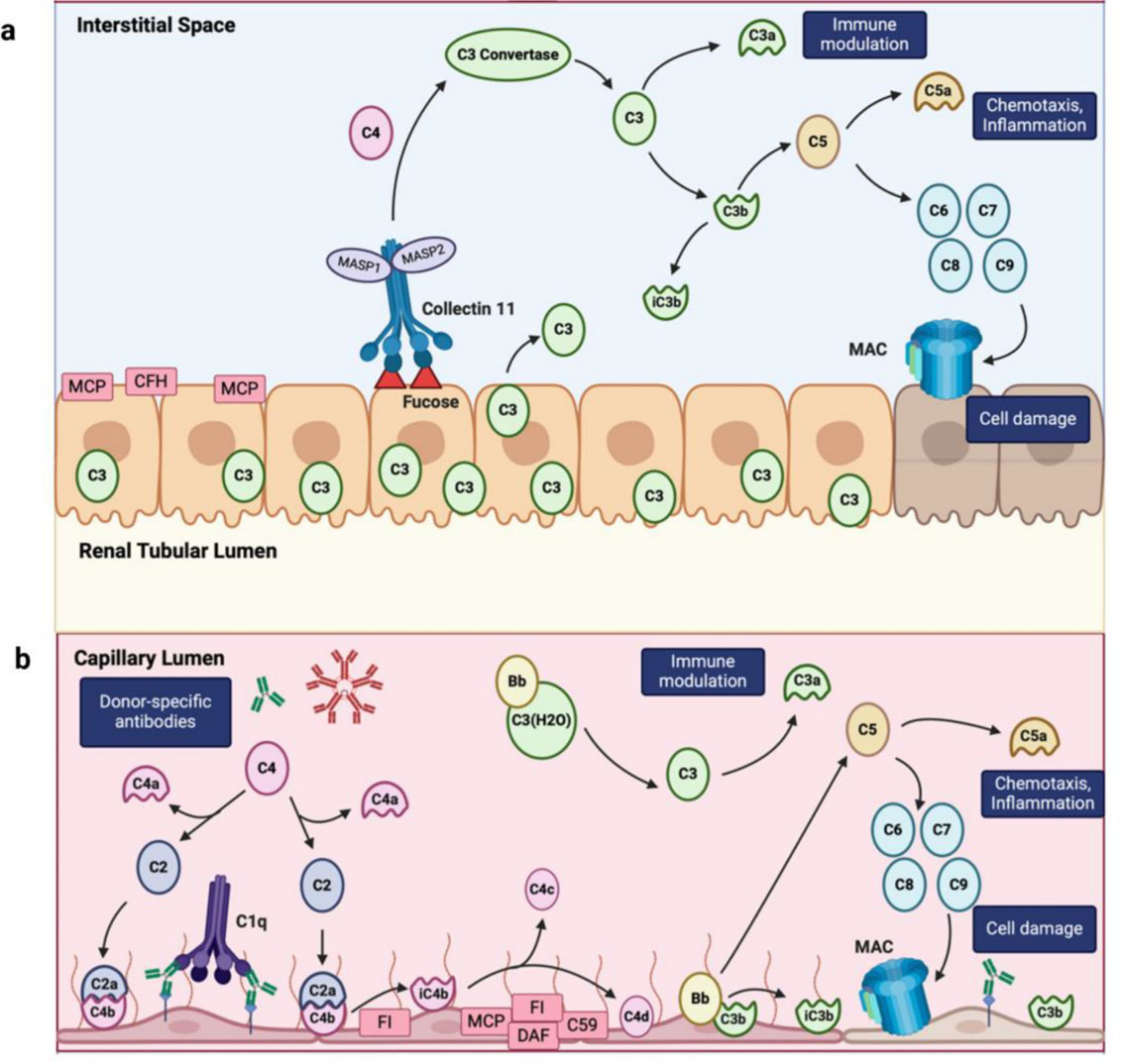
Mechanisms of complement- and antibody-mediated kidney graft rejection. (**a**) Mechanism of complement-mediated tubular injury during IRI. Increased expression of L-fucose and collectin-11 occur on the basolateral membrane of the renal TECs during IRI. Mannan binding lectin serine protease 1 (MASP1) and MASP 2 bind to collectin-11 and activate the complement via the lectin pathway, while the CP does not appear to have a central role. Formation of the MAC leads to TEC damage. C3 production in hypoxic TECs further fuel complement activation, which is uncontrolled by loss of regulatory proteins such as CFH and MCP (CD46) and favors immune cell chemotaxis. (**b**) Intrarenal complement activity during ABMR. Anti-donor-specific HLA (donor-specific antibodies) and anti-ABO antibodies, mostly IgM and rarely IgG bind to endothelial cells, leading to the classical complement pathway activation. Formation of C3 convertase also causes activation of the alternative complement pathway. Complement regulators on the endothelial cells such as DAF (CD55), MCP (CD46), Factor H, and Factor I limit activation of the AP, but endothelial injury leads to their reduced expression. As a result, further complement activation leads to the recruitment of immune cells and direct endothelial cell injury. Additionally, the CP produces an activated split product (C4d), which shows great potential as a marker for ABMR [110].

Collectin 11 (CL-11) is a member of the lectin family of pattern recognition molecules. It has antimicrobial functions and can activate complement via the LP [63]. In mouse models of IRI, CL-11 is produced by TECs and its formation increases rapidly in the post-ischemic period. Under these conditions, L-fucose, the preferred monosaccharide recognized by CL-11, and complement deposits co-localized with CL-11 along the basolateral surface of the proximal renal tubule [64]. The interaction between CL-11 and L-fucose was further confirmed in a mouse model in which administration of exogenous L-fucose, used to saturate CL-11, prevented complement activation and acute post-ischemic kidney injury. Administration of a second bolus after induction provided additional protection to the kidney. CL-11 knockout mice did not receive additional protection from L-fucose administration, suggesting that the mechanism of L-fucose therapy is largely CL-11 dependent [65] (Fig. 4a). Although the AP plays a significant role in IRI, these new data demonstrate that CL-11 and the MBL pathway are also important for triggering complement activation in the setting of IRI.

#### Kidney complement production and graft rejection

Analysis of renal allograft biopsies revealed C3 mRNA and protein in the glomeruli and tubules. This data suggests that donor-derived C3 synthesis within the TECs contributes to graft injury [66]. The amount of mRNA encoding components of the AP and their receptors are also higher in human allograft biopsies with histological evidence of rejection compared to healthy control tissue [54, 67, 68]. Interestingly, different C3 haplotypes and their expression levels in donor kidney cells may also influence long-term graft outcomes. Humans express two common C3 allotypes, slow and fast (C3S and C3F, respectively), so named based on electrophoretic mobility. Brown *et al.* showed that donor organs carrying at least one allele of the C3F variant had better function at 5 years post-transplant (∼10 ml/min greater eGFR) than C3S/S homozygote kidneys transplanted into recipients that were homozygous for the C3S allele. In this cohort, there was no difference in rejection between mismatched donor and recipient C3 allotypes [69]. The protective mechanism of this finding remains unclear but is strongly supportive of a functional role of complement in post-transplant graft injury.

Altogether, these findings underscore the potential impact of complement genetic variations on transplant outcomes. Incorporating donor-recipient complement genotype matching into clinical practice could enhance risk stratification and personalize immunosuppressive strategies. However, further large-scale, prospective studies are necessary to validate these associations and to assess the feasibility and cost-effectiveness of routine complement genotyping in optimizing transplant success.

### Kidney complement production and humoral rejection

In the Banff 2022 classification [70], peritubular capillary C4d deposition is a diagnostic criterion for antibody-mediated rejection (AMR). Interestingly, incubation of endothelial cell lines with HLA-specific antibodies resulted in a significant increase in C4 protein synthesis [71]. Additionally, human kidney transplants with histological evidence of chronic AMR demonstrated an increase in local C3 expression. In these patients, the downregulation of intrarenal complement regulatory proteins was associated with poorer long-term graft outcomes [72]. Together, the data support a role for kidney-derived complement in the severity and progression of AMR.

### Kidney complement production and cellular rejection

Intrarenal complement production may also play a primary role in mediating ACR. It is well established that immune cell-derived complement enhances effector T-cell differentiation and survival [54, 73–75]. Using mouse models, Pratt *et al.* confirmed increased C3 gene expression in renal allografts during acute ACR and demonstrated its significance in the pathogenesis of ACR [41]. C3-deficient mice that received a renal allograft from WT mice developed rapid graft rejection. In contrast, WT recipients of C3-deficient allografts did not develop acute rejection and had significantly improved graft survival, suggesting a dominant effect of graft-derived complement in this model.

*In vitro* and *in vivo* experiments demonstrated local complement production primed the effector CD8 T-cell response to allogeneic vascular endothelial cells through C5a–C5aR1 interactions [67, 76, 77]. Moreover, anti-C5 mAb synergizes with CTLA4-Ig to prevent T-cell priming and trafficking to the allograft, which in turn prolongs graft survival in mice [78]. In summary, complement is required to induce and amplify the T-cell alloimmune response and the renal allograft itself can sustain this pathogenic cycle in both animal models and humans (Fig. 4b).

Complement is also essential for priming of dendritic cells (DCs). Different subsets of human DCs produce complement, and C5aR/C3aR signaling regulates DC activation and function [79]. DCs deficient in C3 have a reduced capacity for T-cell stimulation, resulting in impaired allograft recognition by the recipient immune system [80]. *In vitro*, human T cells responding to allogeneic DCs produce C3a and C5a [79]. Both T cells and DC express the receptors for C3a and C5a. C3aR and C5aR antagonists inhibit human T-cell proliferation, while recombinant C3a/C5a promotes the expansion of alloreactive human CD4 T cells [81–83]. Also, the downregulation of complement regulator DAF on DCs is associated with increased C3a and C5a production [79]. WT mice reject DAF-deficient allografts with accelerated kinetics due to complement-dependent augmentation of anti-donor T-cell immunity [84], sometimes even bypassing the requirement for T-helper cells [85]. Local complement production can alter the ability of donor antigen-presenting cells to prime the antigen-specific T cells that mediate rejection [41, 74, 86, 87]. These cells, also known as donor passenger cells, reside specifically around the renal tubules and can migrate into the recipient's lymphoid system where they immunize the recipient against donor major histocompatibility complex antigens [88].

### Intracellular complement: the complosome

**Figure 5:**
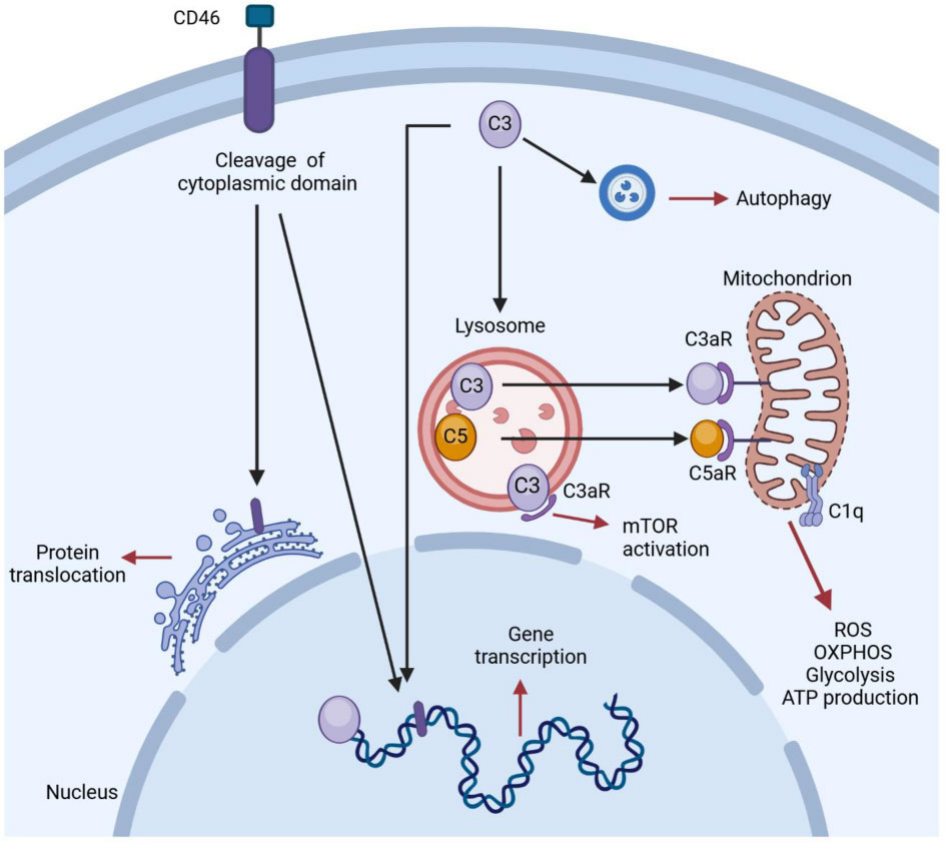
Overview of intracellular “complosome” and effects on cellular physiology. There are different subcellular locations for complosome activation, from the cell membrane to lysosomes and mitochondria, and its proteins can also condition intranuclear gene transcription. Both the cytoplasmic domain of CD46 and C3 itself augments gene transcription of effector molecules in the nucleus, including IFN-gamma and granzyme. Lysosomal cleavage of C3 and C5 generates C3a and C5a, respectively. Ligation of C3a to lysosome-expressed C3aR activates mTOR signaling to promote cell survival and regulate cell growth. The C3a and C5a can also promote mitochondrial activity by binding to their respective receptors on the mitochondrial surface, stimulating activation of numerous pathways (some of which are canonically associated with inflammation), such as: glycolysis, production of reactive oxygen species, oxidative phosphorylation (OXPHOS), ATP production. The C1q bound to mitochondria seems to reduce glycolysis during infection to protect the tissues [91].

Complement activation and effector functions occur separately in the extracellular and intracellular space in a variety of cell populations and tissues. The intracellularly active complement system is termed the “complosome” [89, 90]. Its core components, C3 and C5, along with their receptors, are found in multiple intracellular locations, including the cytoplasm, lysosomes, endoplasmic reticulum, outer mitochondrial membrane, and nucleus [91]. The complosome is actively involved in the regulation of fundamental cellular processes such as metabolism, autophagy, and gene expression [92, 93]. Perturbations in complosome activities are associated with several human diseases, including recurrent infections, arthritis, atherosclerosis, and cancer [94, 95]. In the kidney, the complosome plays a crucial role in maintaining endothelial and epithelial cell integrity and function. The recent identification of the complosome has sparked growing interest in further research to better understand its mechanisms and develop approaches to target it therapeutically [91] (Fig. 5).

#### The complosome in kidney cells

Emerging data suggest that dysregulation of the complosome plays an important role in kidney disease pathophysiology [96]. Changes in the intracellular activities of complement components in kidney endothelial or epithelial cells contribute to organ dysfunction and fibrosis after kidney injury [97]. The absence of intracellular CFH led to spontaneous cytoskeletal remodeling and aberrant cell layer integrity, resulting in changes in cell morphology and the loss of barrier function. Additionally, alterations in mitochondrial respiration and ATP production leading to increased cell proliferation and increased angiogenic potential were also described [97]. Interestingly, while glomerular endothelial cells produce CFH, TECs at steady state have undetectable intracellular CFH levels. Accordingly, CFH in TECs knockdown had no major effect on their function or transcriptional program [98].

#### The complosome in renal immune infiltrates

Intracellular complement components play diverse roles in modulating immune responses in various immune cell types. In human CD4^+^ T cells, intracellular C3 stores contribute to homeostatic survival via mTOR activation and are essential for inducing T-helper 1 (T_H_1) effector responses. Antigen binding and CD28 engagement induce rapid shuttling of intracellular C3b to the T-cell surface, triggering nuclear translocation of CD46 domains, crucial for IFN-γ production and T_H_1 cell induction [90]. Complement has a critical role in CD8^+^ T-cell responses, modulating cytotoxic activity and immune regulation. Complement activation fragments, particularly C3a and C5a, enhance CD8^+^ T-cell responses by promoting IFN-γ production and cytotoxic function. The engagement of C3aR and C5aR1 on CD8^+^ T cells enhances their survival, proliferation, and cytokine secretion. Intracellular complement components, as part of the complosome, may further influence these T-cell responses by regulating internal cellular processes (signaling, survival, and activation) [99]. Deficiencies in complement components impair CD8^+^ T-cell responses, reducing antiviral and alloimmune defense [94].

Neutrophils, monocytes, and macrophages rely on intracellular C3 and C5 activation for various functions, including cytoskeletal rearrangement, metabolic reprogramming, and inflammasome activation. Intracellular complement activity in macrophages also influences the polarization toward a pro-inflammatory phenotype or non-inflammatory removal of dead cells [100]. Moreover, intracellular complement controls the opsonization of target cells and antigen processing, impacting macrophage-directed T-cell responses. The multifaceted roles of intracellular complement highlight its significance in immune regulation and cellular functions, presenting potential targets for therapeutic interventions in various immune-related conditions.

In a folic acid-induced tubular injury model, intracellular C5 and C5aR1 activity in kidney-infiltrating macrophages appears to contribute to nephropathy. Mice with C5- or C5aR1-deficient macrophages exhibited reduced kidney fibrosis compared to WT controls, and mitochondrial C5aR1 hyperactivation was identified as a key driver of the initial folic acid-induced injury [101, 102]. Furthermore, single-cell RNA sequencing of kidney tissue revealed that unilateral ureteral obstruction in mice increases renal C3 and C5 expression, primarily in TECs, alongside upregulated C3aR and C5aR1 expression in kidney interstitial immune cells [102].

To date, our understanding of the complosome on immune cell function and kidney injury has come from humans with genetic defects in complement genes [103] and various rodent models. There is no clear complosome data in kidney transplantation, but a better understanding of its role may pave the way for future studies targeting the complosome or using it as a biomarker of graft health.

## COMPLEMENT-TARGETING THERAPIES IN KIDNEY TRANSPLANTATION

Major clinical studies describing complement-targeting treatments are listed in Table 1. Eculizumab, a monoclonal antibody that inhibits the cleavage of complement component C5 and prevents the formation of the MAC, has been the most extensively investigated complement inhibitor in kidney transplantation. Initially approved for atypical hemolytic uremic syndrome (aHUS), eculizumab has been used off-label as prophylaxis for AMR in sensitized kidney transplant recipients. Clinical studies have reported reduced rates of early AMR and improved short-term graft survival when eculizumab was used peri-transplant in patients with high levels of donor-specific antibodies [104]. Ravulizumab is an anti-C5 molecule created by replacing four amino acids in the eculizumab molecule to increase the duration of the inhibitory effect. It has been studied for maintenance of aHUS remission post kidney transplant [105], however, large prospective trials are still pending.

**Table 1:** Major clinical trials on complement-targeting treatments in kidney transplantation.

Publication/trial code	Design and treatments	Patient population	Follow-up	Primary endpoint	Outcomes
Kaabak *et al.* [111]/NCT01756508	RCT: eculizumab (*n* = 29) vs. placebo (*n* = 28)	Pediatric (1–18 years old) KT recipients with IRI risk	36 mo	Incidence of DGF	Day 1 eGFR, eculizumab vs. placebo: 82 vs. 49 ml/min/1.73 m^2^; *P* = .01; Day 2 eGFR: 117 vs. 71 ml/min/1.73 m^2^; *P* = .004. No GFR difference at 3 yrs. 4 graft losses due to flu-like illness in the eculizumab group in non-vaccinated patients
Stegall *et al.* [104]/NCT00670774	Open-label, single center: eculizumab (*n* = 26) vs. historic controls (*n* = 51)	KT recipients at risk of ABMR	15 mo	ABMR at 3 mo	ABMR, eculizumab vs. placebo: 7.7% vs. 41.2%; *P* = .0031
Marks *et al.* [112]/NCT01399593	RCT: eculizumab (*n* = 51) vs. SOC (*n* = 51)	Sensitized KT recipients at risk of ABMR	12 mo	Composite of ABMR, graft loss, and death at 9 wks	Composite ABMR, eculizumab vs. SOC: 9.8% vs. 13.7% *P* = .760
Glotz *et al.* [113]/NCT01567085	Open-label, single-arm: eculizumab (*n* = 80)	Sensitized KT recipients at high risk of ABMR	36 mo	Composite of ABMR, graft loss, and death at 9 wks	Composite ABMR, eculizumab vs. expected (historical): 8.8% vs. 40% *P* < .001
Kulkarni *et al.* [114]/NCT01327573	RCT: eculizumab (*n* = 11) vs. placebo (*n* = 5)	KT recipients with chronic ABMR (at least 20% of eGFR decline in 12 mo pre-enrollment)	12 mo	% change in GFR trajectory over 6 mo	Mean GFR, eculizumab vs. control: 30.21 vs. 28.69 ml/min; *P* = .68 Percentage change in GFR, eculizumab vs. control: +5.06% *P* = .09
Vo *et al.* [115]NCT01134510	RCT: eculizumab (*n* = 102) vs. control (*n* = 99)	KT recipients at risk of ABMR	9 wks	Composite: treatment failure (ABMR, graft loss, death)	Treatment failure: 9.8% (eculizumab) vs. 13.7% (control); *P* = .76
–/NCT06830798 [116]	RCT: ravulizumab (*n* = 225 planned) vs. placebo (*n* = 225 planned)	KT recipients at risk of DGF	12 mo	Incidence of DGF	Not yet recruiting
–/NCT04572854 [117]	RCT: pegcetacopan (*n* = 10) vs. placebo (*n* = 3)	KT recipients with clinical and pathological evidence of C3G or IC-MPGN	12 mo	Biopsy-proven C3G recurrence at 12 months	Trial ongoing; no results yet
Vo *et al.* [115]/NCT01134510	RCT: C1-INH (Berinert) (*n* = 10) vs. placebo (*n* = 10)	KT recipients at risk of ABMR	24 mo	ABMR at 1 and 6 months	ABMR, Berinert vs. placebo: 20% vs. 30%; *P* = .6
Jordan *et al.* [118]/NCT02134314	RCT: C1-INH (*n* = 35) vs. placebo (*n* = 35)	KT recipients at risk of DGF	12 mo	Need for hemodialysis during the first week post-transplant	Hemodialysis requirement, C1-INH vs. placebo: 44.12% vs. 60%; *P* = .232
–/NCT04572854 [119]	RCT: efgartimod PH20 for 48 wks (*n* = 10 estimated) vs. efgartimod PH20 for 24 wks followed by placebo for 24 wks (*n* = 10 estimated) vs. placebo for 48 weeks (*n* = 10 estimated)	Sensitized KT recipients at risk of ABMR	18 mo	Incidence of AEs (up to 18 mo), % of participants with permanent treatment discontinuation due to AEs	Recruiting; no results yet
–/NCT05907096 [108]	RCT: empasiprubart (*n* = 68 estimated) vs. placebo (*n* = 34 estimated)	KT recipients at risk of DGF	64 wks	eGFR at 24 weeks post-transplant	Recruiting; no results yet

AEs, adverse events C1-INH, C1 inhibitor C3G, C3 glomerulopathy DGF, delayed graft function IC-MPGN, primary immune complex membranoproliferative glomerulonephritis KT, kidney transplant mo, months RCT, randomized controlled trial; SOC, standard of care, wks, weeks.

Beyond C5 blockade, several novel agents targeting different components of the complement cascade are under investigation. Pegcetacoplan, a C3 inhibitor, offers broader inhibition of complement and is currently being evaluated in clinical trials for transplant and complement-mediated kidney diseases [106], and empasiprubart, a C2 inhibitor, is being tested for efficacy in reducing delayed graft function after kidney transplant [107, 108] and possibly in the future for AMR as the ongoing experimentation is still *in vitro* [107]. Efgartigimod is an IgG1 Fc fragment that targets neonatal Fc receptor. This interaction inhibits IgG recycling and increases its degradation [109].

Despite the therapeutic promise of complement inhibitors, outcomes have been underwhelming in transplant. One particular challenge has been the lack of reliable biomarkers for real-time monitoring of complement activation, which limits the ability to tailor therapy effectively. Given the risk of opportunistic infections with over-immunosuppression, determining a dose that provides clinical equipoise in human patients is a continual consideration. Additionally, since complement is also important for cellular homeostasis, determining the optimal timing, duration, and disease selection for therapy remains a persistent obstacle. Nevertheless, the integration of complement-targeting strategies into transplant immunosuppression protocols holds potential to advance personalized approaches and improve outcomes in high-risk kidney transplant recipients.

## CONCLUSION

In kidney transplantation, renal complement production and activation critically mediate IRI, cellular and humoral alloimmunity, and progressive renal damage. While the role of extracellular complement is well established in post-transplant graft injury, further research is needed to delineate how intracellular complement contributes to graft homeostasis or dysfunction. Substantial challenges remain in terms of monitoring effective complement inhibition, patient selection, and selecting appropriate components of the pathway to target. Ongoing research to delineate how the complement cascade mediates graft injury and/or repair should continue to permit optimization of our treatment strategies to improve care for kidney transplant recipients.

## Data Availability

No new data were generated or analyzed in support of this work. All the sources for this review are cited in the ‘References’ section.
